# Not Drug-like, but Like Drugs: Cnidaria Natural Products

**DOI:** 10.3390/md20010042

**Published:** 2021-12-30

**Authors:** Claire Laguionie-Marchais, A. Louise Allcock, Bill J. Baker, Ellie-Ann Conneely, Sarah G. Dietrick, Fiona Kearns, Kate McKeever, Ryan M. Young, Connor A. Sierra, Sylvia Soldatou, H. Lee Woodcock, Mark P. Johnson

**Affiliations:** 1 School of Natural Sciences and Ryan Institute, National University of Ireland Galway, H91 TK33 Galway, Ireland; claire.laguionie-marchais@nuigalway.ie (C.L.-M.); louise.allcock@nuigalway.ie (A.L.A.); ellie.conneely@nuigalway.ie (E.-A.C.); kate.mckeever@ucdconnect.ie (K.M.); ryan.young@nuigalway.ie (R.M.Y.); 2 Department of Chemistry, University of South Florida, Tampa, FL 33620-5250, USA; bjbaker@usf.edu (B.J.B.); sdietrick@usf.edu (S.G.D.); fionakearns@usf.edu (F.K.); csierra@usf.edu (C.A.S.); sylvia.soldatou2@abdn.ac.uk (S.S.); hlw@usf.edu (H.L.W.); 3 School of Chemistry, National University of Ireland Galway, H91 TK33 Galway, Ireland

**Keywords:** ADME, chemical space, drug-likeness, MarinLit, multivariate, rule-of-five, virtual screening

## Abstract

Phylum Cnidaria has been an excellent source of natural products, with thousands of metabolites identified. Many of these have not been screened in bioassays. The aim of this study was to explore the potential of 5600 Cnidaria natural products (after excluding those known to derive from microbial symbionts), using a systematic approach based on chemical space, drug-likeness, predicted toxicity, and virtual screens. Previous drug-likeness measures: the rule-of-five, quantitative estimate of drug-likeness (QED), and relative drug likelihoods (RDL) are based on a relatively small number of molecular properties. We augmented this approach using reference drug and toxin data sets defined for 51 predicted molecular properties. Cnidaria natural products overlap with drugs and toxins in this chemical space, although a multivariate test suggests that there are some differences between the groups. In terms of the established drug-likeness measures, Cnidaria natural products have generally lower QED and RDL scores than drugs, with a higher prevalence of metabolites that exceed at least one rule-of-five threshold. An index of drug-likeness that includes predicted toxicity (ADMET-score), however, found that Cnidaria natural products were more favourable than drugs. A measure of the distance of individual Cnidaria natural products to the centre of the drug distribution in multivariate chemical space was related to RDL, ADMET-score, and the number of rule-of-five exceptions. This multivariate similarity measure was negatively correlated with the QED score for the same metabolite, suggesting that the different approaches capture different aspects of the drug-likeness of individual metabolites. The contrasting of different drug similarity measures can help summarise the range of drug potential in the Cnidaria natural product data set. The most favourable metabolites were around 210–265 Da, quite often sesquiterpenes, with a moderate degree of complexity. Virtual screening against cancer-relevant targets found wide evidence of affinities, with Glide scores <−7 in 19% of the Cnidaria natural products.

## 1. Introduction

The diversity of natural products presents a problem: the metabolites have potential in many therapeutic areas, but where does the potential lie? One route to defining applications for natural products is through bioassays. However, comprehensive bioassays are resource intensive. There is therefore a need to characterise natural product databases to identify molecules with properties that may be of value for development. In this study, we focus on Cnidaria-derived natural products (NPs). Phylum Cnidaria (broadly, corals, anemones, sea pens, and jellyfish) is of interest as the second largest source of marine natural products from invertebrates (30% compared to 46% from Porifera [1]). The sources of natural products in both Porifera (sponges) and Cnidaria may, however, have a symbiont origin. Various studies have shown associated microorganisms (Eukarya, Archaea, Bacteria) to be the producers of many NPs [2,3]. Our current study excluded NPs known to have a symbiont origin. Cnidaria NPs are chemically diverse, and include toxins and venoms, terpenoids, diterpenes, prostaglandins, and steroids [4,5]. Where assays have been carried out, these have demonstrated cnidarian NPs with antibacterial, cytotoxic, anticancer, anti-inflammatory, and antiviral properties [1,4,5,6]. Despite the potential of Cnidaria NPs, they are a poorly characterised group. The marine natural product (NP) database MarinLit (http://pubs.rsc.org/marinlit, accessed in December 2018) has 5827 metabolites isolated from Cnidaria published in 1828 papers. By randomly taking 100 papers spread over the full period of records (1940–2018), we estimated that only 24% of papers screened one or several metabolites against one or a few human diseases. This implies that Cnidaria NP potential bioactivity is under-explored.

There are various routes to evaluate the potential of natural products prior to undertaking targeted assays. These include comparing NPs with existing drugs, making comparisons with ligands of interest, virtual screening against potential targets, and making predictions from structure–activity relationships. When NPs are compared with existing drugs, these comparisons often use the feature characteristics of existing drugs to define ranges of suitable molecular properties (a drug-likeness approach, e.g., [7,8]). An expansion of the drug likeness approach is to review the relative positions of natural products and other compounds in chemical space (e.g., [9,10]). There are implications to using approved drugs as reference data. The population of approved drugs represents many steps beyond basic efficacy, involving processes such as commercial decisions, luck, and changes in scientific focus. This means that opportunities may be missed if they are not represented in the drugs that have made it through the various filters to approval. As an alternative to looking at existing drugs as a reference space, NPs can be screened using similarity to known ligands. Direct estimation of ligand suitability by virtual screening of metabolites against drug targets can also be used to prioritise NPs of interest. A very flexible approach is to define a desired biological function for a compound and to use quantitative structure–activity relationships (QSAR) to quantify how molecular properties affect degree to which a compound displays this function. The uses of QSAR in drug discovery include screening for specific activities or more general properties like the blood-brain barrier permeability or toxicity of different NPs. 

The application of different methods generally addresses different facets of the potential value of NPs. Contrasts between methods can therefore be more informative than the application of a single approach. The concept of ‘value’ is, of course, multifaceted and depends on the application. We are interested in the general characterisation of a large taxonomically-defined group of marine NPs. In this study, we apply examples of drug-likeness (QED, RDL, and rule-of-five), ligand likeness, molecular docking, and toxicity QSAR (ADMET-score) to define NP potential and provide a synthesis for metabolites from Cnidaria. Cnidaria NPs were compared to both drug and toxin reference sets using 51 properties, including absorption, distribution, metabolism, and excretion (ADME) variables derived using the Schrödinger QikProp software.

## 2. Results

**Figure 1:**
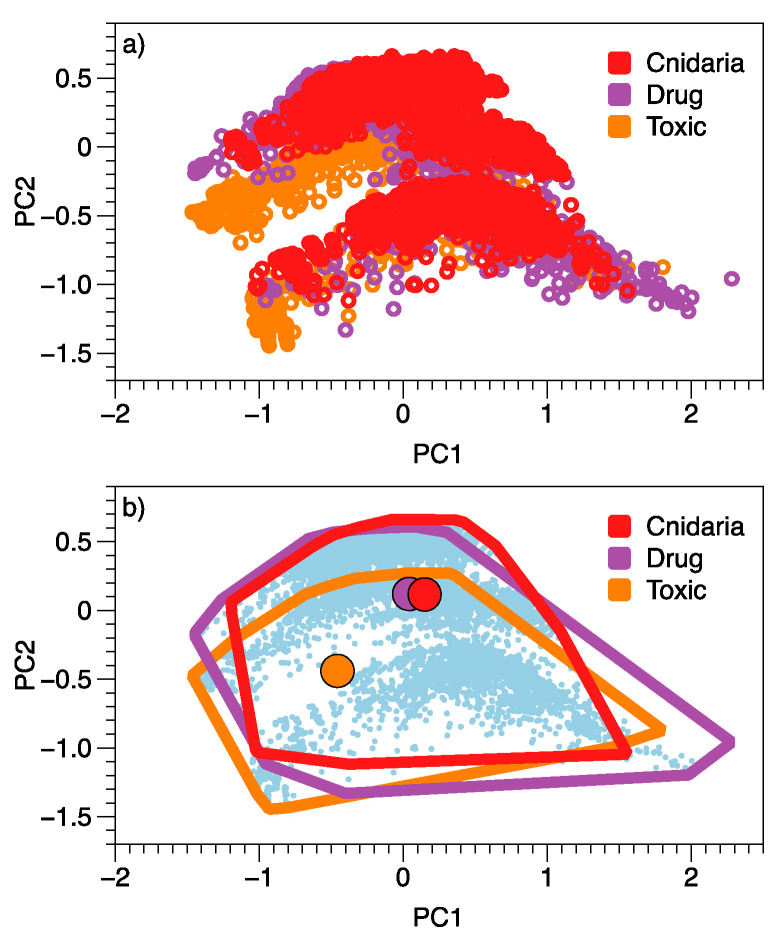
Relationships among approved drugs, Cnidaria NPs and toxic compounds based on a principal components analysis of 51 QikProp-generated variables for each molecule. Group membership for individual molecules is highlighted in (**a**), with centroids and outliers of each group emphasised in (**b**).

Cnidaria NPs (*n* = 5600), drugs (*n* = 2009) and a reference set of active, non-drug molecules (‘toxic’, *n* = 2012) do not appear to be particularly separate in the first two dimensions of a PCA based on QikProp generated properties (Figure 1). The centre of the drug and cnidarian point clouds are closer to each other than either are to the centre of the toxin points. The outer ranges of the different sets of molecules, however, largely overlap (Figure 1b). The two axes of the PCA (PC1 and PC2) account for 58.9% of the overall variation among molecular Qikprop properties. Molecules with higher predicted central nervous system activity (CNS), higher predicted apparent Caco-2 cell permeability (QPPCaco, a model for the gut-blood barrier), higher predicted apparent MDCK cell permeability (QPPMDCK, a model for the blood-brain barrier), and better human oral absorption tend to be towards the left side of the horizontal axis: PC1 (Table 1. These predictors have negative correlations, so have high values when PC1 values are negative). Variables associated with more positive values along PC1 are the number of violations of Lipinski’s rule-of-five, the number of heavy atoms (X.nonHatm, linked to metabolism) and the predicted polarisability (QPpolrz). The strongest influence on molecule position along PC2 was oral absorption.

Despite the overlap in two dimensions (Figure 1), there were differences in the location and/or spread of the cloud of points for each category (Permanova, *n* = 3, *r*^2^ = 0.15, *p* = 0.001, *F*_2,9618_ = 834.5).

**Figure 2:**
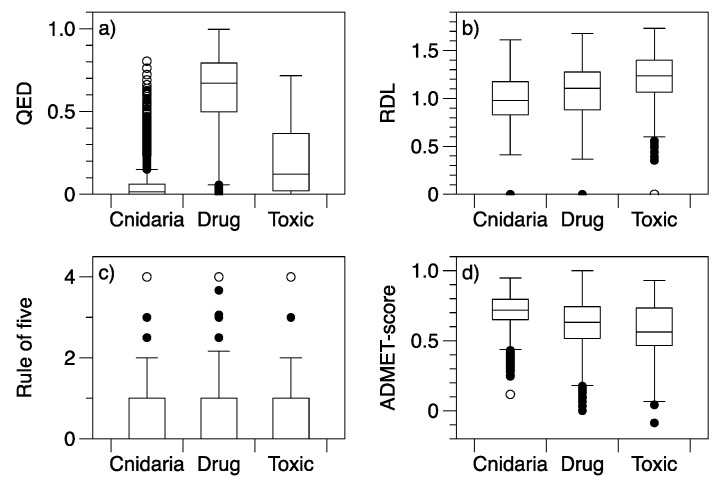
Boxplots of drug-likeness indices and ADMET-score for drug, Cnidaria, and toxic compounds: (**a**) QED, (**b**) RDL, (**c**) Rule-of-five violations (Ro5) and (**d**) ADMET-Score. All drug-likeness scores are standardised to a 0–1 range, with higher QED, RDL, and ADMET-score indicating molecules with more drug-like properties.

The differences among compound categories in multivariate space are reflected by differences in drug-likeness scores. All drug-likeness scores and the ADMET-score had some differences among groups (Table 2). Not surprisingly, drugs generally had higher QED scores than toxins or Cnidaria NPs (Figure 2). The use of the number of structural alerts in QED and RDL was not sufficient for the toxin group to receive relatively poor scores, the toxins had more favourable scores than Cnidaria NPs in both cases. The ADMET-scores were generally higher (more favourable) for Cnidaria NPs than the other groups, indicating low predicted toxicity. As would be expected, the toxin group had the lowest median ADMET-score, reflecting higher predicted toxicities. The ADMET-score was therefore informative in identifying harmful properties that were not identifiable from the variables used to define the RDL. Data for the rule-of-five (Ro5) have too few categories for a box plot to be particularly informative for comparisons, but the proportion of molecules where at least one threshold was exceeded was greater for Cnidaria NPs and toxins (37% and 39% of molecules with at least one variable outside the Ro5 thresholds) than it was for drugs (26% of molecules with at least one exception to the Ro5). 

Some drug-likeness indices were correlated, reflecting similar information being used to generate the value (e.g., QED and RDL, overall correlation using drug, NP and toxin data = 0.355, *p* < 0.05). Measures of chemical space similarity between Cnidaria NPs and drugs also tended to show correlations, but the patterns were more complex (Table 3). The Jaccard score for the similarity of a Cnidaria NP to drugs was correlated to the NP’s RDL and ADMET-score. However, this measure of apparent drug-likeness was not reflected by the corresponding QED score, with a weak, but significant, negative correlation. The negative correlation of Jaccard score and Ro5 is less surprising: higher Jaccard values indicate a metabolite closer to the centre of the cloud of drugs and with a lower frequency of Ro5 threshold exceedance. The calculated Jaccard coefficient also reflects the spread of points in the PCA, with more drug-similar metabolites to the left of PC1 and higher on PC2. The Tanimoto score summarises different information about the Cnidaria NP, although this is not so easy to interpret as the correlations are relatively weak, even when significant.

The screen of Cnidaria metabolites using BindingDB identified over 17 thousand potential targets, with a maximum of 51 for a single NP, but 2730 metabolites in the NP dataset with no identified target. The number of BindingDB hits for a metabolite was weakly correlated with the metabolite’s Jaccard, Tanimoto, RDL, ADMET-score, and Ro5 values. While these relationships were significant, they were weak and negative for Jaccard, Tanimoto, and RDL (range −0.097 to −0.057). The number of BindingDB hits that a coral metabolite had were positively correlated with the ADMET-score and Ro5, but correlations were weak: below 0.166. Glide screening also identified potential ligands for proteins of interest, with 1530 ‘conformational hits’ (Glide score < −7) from 1076 (19%) of the Cnidaria NPs.

The various indices summarise different aspects of a Cnidaria NP’s potential value. They also emphasise that a single index, such as the QED, may not give a full picture (e.g., the best QED values are from drugs, but drugs are generally less favourable considering the ADMET-score). One way to identify the most favourable metabolites is to combine different indices. To do this, values were standardised by subtracting values from the index mean and dividing by the standard deviation. This reduces the scope for a single index to dominate the response. A summed index was then calculated using the Jaccard, Tanimoto, RDL, ADMET-score, and (-Ro5). The number of BindingDB hits was omitted from the summed index due to the weak correlation with other indices and the large number of zeros. All the included index values are positively correlated with the Jaccard index and indicate generally drug-like properties. Note that multiplying the standardised Ro5 score by −1 means that positive values represent fewer Lipinski threshold exceedances and therefore more drug-like properties. QED was left out of the summed index. The weaker negative correlation of QED with the Jaccard coefficient (Table 3), indicates that these measures may contain different information about metabolite drug likeness. The post-treatment summation of indices therefore left two scores to evaluate Cnidaria NPs: a summed index (mean 0, SD 3.23) and QED.

**Figure 3:**
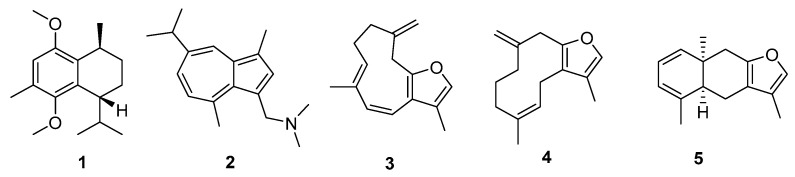
Cnidaria NPs with relatively high QED (>0.55) and summed drug index values (>2.42). Unique identifiers in MarinLit and names (where a name was used in the original description) are as follows: (**1**) L22017, (**2**) L1211 N,N-dimethylamino-3-guaiazulenylmethane, (**3**) L1854 furanotriene, (**4**) L339, (**5**) L2401 tubipofuran. Three of the metabolites had at least one Glide score more negative than −7.

The tails of the summed index and QED distributions can be used to indicate Cnidaria NPs with consistent drug-likeness, NPs with drug-likeness in one index and not the other, and NPs that are consistently least like drugs. The most consistently druglike NPs are those with a high summed drug-likeness index and a high QED. Cnidarian metabolites with these properties included sesquiterpenes and a nitrogenous azulene derivative (Figure 3). Only one of the source description papers reported bioactivity (barnacle cyprid inhibition and *Artemia* nauplii mortality in **1**, [11]). This pattern of bioactivity is consistent with a view of sesquiterpenes as defensive metabolites, also reflected in activity against *Vibrio harveyi* [12]. The docking scores from Glide indicate that three of the favourably ranked metabolites could quite possibly bind to target proteins, with five hits overall. Metabolite **2** had a score of −8.7 with a sex hormone binding globulin (UniProID PO4278). Metabolite **3** had scores of −7.6 and −7.0 with the sex hormone binding globulin and serum albumin respectively. There were also Glide hits for **4**: −8.5 for sex hormone binding globulin and −8.2 for the G-protein coupled estrogen receptor.

**Figure 4:**
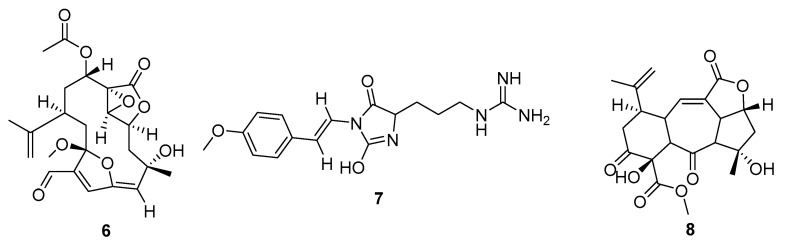
Metabolites from Cnidaria with poor QED scores (=0), but favourable summed drug similarity indices (>2.06). MarinLit codes and names are: (**6**) L12916 3-methoxy-8-hydroxylophotoxin, (**7**) L27157 parazoanthine F, (**8**) L24340 sinumaximol I.

The key molecular properties of the QED were chosen for oral bioavailability and to reduce the chances of a drug having undesirable properties [13]. The molecules with higher summed drug index scores, but poor QED values reflect this (Figure 4). The metabolites are noticeably larger and more complex than those in Figure 3. The original descriptions noted a chemical defence activity against grazing fish for **6**, L12916. The parazoanthines (e.g., **7**) are a series of metabolites that have been identified as agonists of the CXCR4 chemokine receptor [14]. Potential cancer target activity was also reflected by the docking in the current study, with a glide score of −10.2 against Tyrosine-protein kinase ABL1 for one of the parazoanthines.

**Figure 5:**
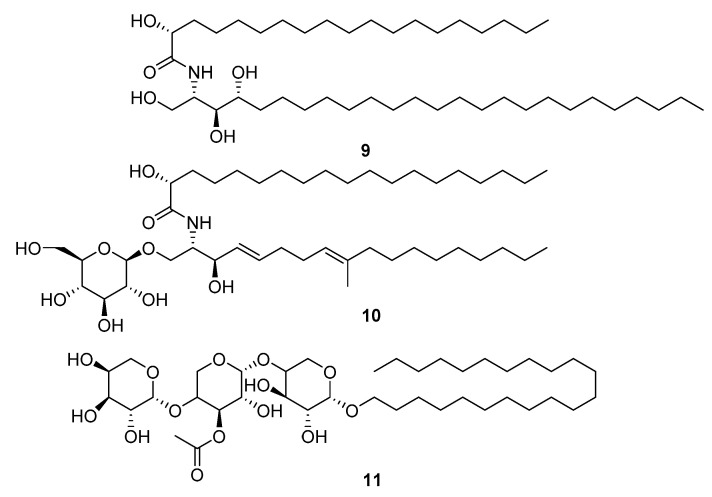
Cnidaria NPs with low QED (=0) and negative (unfavourable) summed drug similarity scores (<−12.94). MarinLit information: (**9**) L24815, (**10**) L15811, (**11**) L17422 firmacoside B.

The Cnidaria NPs ranked as least druglike by summed index and QED were relatively large metabolites: glycosphingolipids, ceramides, sarcoehrenosides, glycosides, and sphingolipids (Figure 5). This group was not without activity in the original descriptions of the molecules. Metabolite **9** (L24815) was shown to have some activity against H5N1 avian influenza [15]. None of this group had activity in the virtual docking screening with Glide.

**Figure 6:**
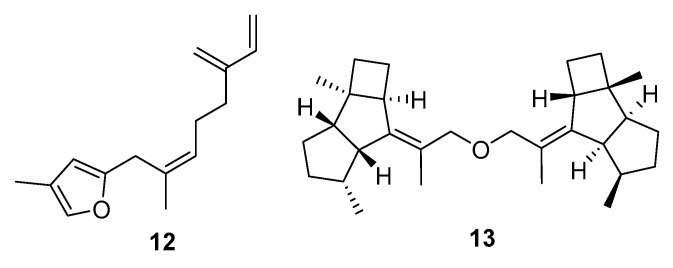
Cnidaria NPs with negative (unfavourable) averaged drug similarity scores (<−2.6), but higher QED values (>0.53). MarinLit references are: (**12**) L1250 and (**13**) L25758 dikelsoenyl ether.

A small group of molecules had QED scores above 0.5 but relatively low summed index values (Figure 6). The majority of the metabolites were furanosesquiterpenes (e.g., **12**), with one dikelsoenyl ether (**13**). The good QED scores presumably reflect good solubility and hydrogen donor/acceptor properties. No activities were reported in the original articles describing these metabolites. There were also no affinities identified in the virtual docking carried out using Glide. 

## 3. Discussion

Cnidaria are clearly a source of many potentially useful drugs and drug leads. The Cnidaria NPs superficially occupy a similar chemical space to drugs and toxins, based on ADME and molecular properties. Multivariate analysis, however, indicates that the groups can be distinguished, with category (NP, drug, or toxin) explaining 15% of the variation among molecules. In terms of indicators based on eight or fewer molecular properties (QED, RDL, Ro5), many Cnidaria NPs do not have particularly drug-like properties. However, in terms of predicted toxicity (ADMET-score) Cnidaria NPs are generally more favourable than drugs or the toxin reference set. When the ADMET-score was created [16], toxicity was disassociated from the ADME properties, showing no difference in overall ADME properties between approved and withdrawn drugs but a significant difference in terms of toxicity between them. This implies that information on toxicity is independent of other information about the properties of drug candidates [16]. Monitoring a compound’s ADMET problems early on in drug development is a powerful optimisation strategy [17]. Cnidaria metabolites had a similar level of carcinogenicity to drugs and better results in terms of Ames mutagenicity (carcinogenicity and intrauterine toxicity), acute oral toxicity, and human ether-a-go-go-related gene inhibition (hERG, cardiovascular toxicity). In the ADMET sense, Cnidaria metabolites are good starting points for drug development.

A relatively poorer performance for Cnidaria NPs in QED, RDL, and Ro5 is not surprising as these indices were not derived with biologically-sourced molecules in mind. Many, if not all, of the biologically sourced molecules are under evolutionary selection pressure to be active in living systems, so may have features that make thresholds affecting synthetic molecules redundant [18]. These drug likeness indices do, however, allow both individual metabolites and the whole cnidarian library to be compared to the idealised properties of established drugs.

When considering just the Cnidaria NPs, the different measures of drug-likeness or similarity are not all aligned: they emphasise different information. For example, close proximity to the centre of drugs in the QikProp variable space (measured by Jaccard similarity) is not correlated to a Cnidaria NP’s QED score. A weak correlation (0.12) between ADMET-score and QED was also noted for the set of drugs and toxins analysed by [16]. Using combinations of indices (e.g., Figure 3, Figure 4, Figure 5 and Figure 6) can therefore identify coherent groups of drugs for different purposes and give a fuller exploration of potentially useful chemical space than reliance on one index alone. A similar PCA approach [19] also emphasised areas in the expanded chemical space occupied by NP-derived drugs compared with the more narrowly defined synthetic drug space. 

**Figure 7:**
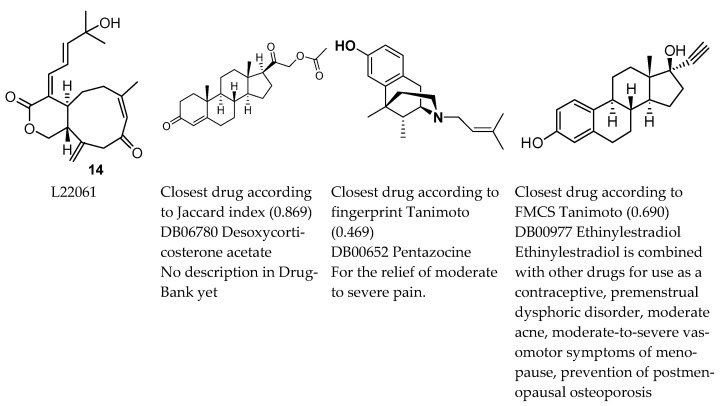
Closest drugs to **14** (L22061, asterolaurin H), a xenicane diterpenoid (Lin et al., 2011 [29]). This metabolite had relatively high Jaccard and Tanimoto values with respect to approved drugs, but average QED, RDL, and Admet scores. Lin et al., 2011 [29] reported cytotoxic activity against Hep-2 (human laryngeal carcinoma), Daoy (human medulloblastoma), MCF-7 (human breast adenocarcinoma), and WiDr (human colon adenocarcinoma) tumour cells.

The use of drug-likeness or drug-similarity measures implies that it is good to be similar to an existing drug. The Ro5 has been a useful reference point [20] despite some exceptions among drugs; however, there is no absolute value for the similarity between a pair of molecules that could be a universal decision point; in addition similarity measures are dependent on representation and context [21,22]. An example of similarity pairs is given in Figure 7. Quite different drugs are selected with different similarity measures. One might want to distinguish between physico-chemical and structural properties when considering whether similarity to a drug is desirable or not [23]. Structural similarity has been a pillar of drug discovery and is based on the observation that structurally similar molecules tend to have similar properties [21]. If a structure binds to a target, then small structural change to this compound should retain biological activity against this target [24]. However, activity cliffs occur [22,25]. From a structural point of view, both similarity (for example, privileged structure, [26]) and novelty (for example, novel building blocks, [27]) have been praised, therefore being similar to a drug can be positive or negative. Being similar or not may be relevant for understanding side effects. Alternatively, drug similarity may identify proposals for therapeutic use on multiple targets, or new uses for existing drugs (polypharmacology and repurposing, [22,28]).

The question of desirability of similarity is less problematic for physico-chemical properties. Physico-chemical property similarity has also guided the development of numerous drugs. For example, the rule-of-five was built on the notion of shared properties among oral drugs [7]. However, such rules with hard filters are subject to scepticism [30]. Trying to be similar to known drugs may have caused a reluctance to explore novel chemical space where important drug discovery opportunities may exist [31]. Some targets are classified as difficult to drug as they have binding sites that are large, highly lipophilic or polar, flexible, flat, or featureless [31] and so have poor affinity with drugs following Lipinski’s rule. Thus, future drugs may be very different from existing examples, with expanded ranges of physico-chemical properties and more non-oral drugs. Balancing advances in the sort of molecule that may become a drug are the limits of what works in humans. Therefore, future drugs will still need to follow some administration, distribution, metabolism and excretion rules (even if these are not defined as yet), which may restrict the degree of variability in physico-chemical properties compared to structural properties.

The relative under-exploration of Cnidaria NPs in assays does not appear to be related to the degree of drug-likeness for any particular molecule. For example, the theoretically most favourable molecules (Figure 3) were not noticeably more assayed in their original descriptions compared to other Cnidaria NPs. Follow-up assay papers were rare, with most citations of original descriptions relating to descriptions of similar compounds or consisting of reviews. For example, [29] (original description of **14**) has been cited seven times in Web of Science to date, with four reviews or general citations, two papers on the same source organisms and one study describing a novel, related, metabolite ([32], L28930 in MarinLit). 

The contrasting of different drug-likeness and similarity measures may be a tool to direct future assay activity, for example, by identifying metabolites that are in the drug-like physico-chemical space, but with less structural similarity (implying more chance of novelty). Alternatively, bioassays could be targeted across a gradient of drug-likeness values, while holding another property in a narrow range, to explore potential activity. These studies can be combined with virtual screens. In the current study, we were able to identify candidate ligands for cancer-related proteins. The chance of a Glide hit was higher in the most favourable NPs (Figure 3, 3/5 molecules with at least one Glide score <−7) compared to low summed index metabolites (Figure 5 and Figure 6, no Glide scores <−7). Overall, our conclusions are similar to those made by [33] in respect of traditional Chinese medicines: Cnidaria NPs have good drug-likeness profiles and provide a diverse range of metabolites for future exploration.

## 4. Materials and Methods

### 4.1. Data Collection and Variable Generation

The study is based on a Cnidaria natural product dataset but involves comparisons of likeness with approved drugs. Furthermore, a toxicant dataset was used as alternative point of reference, to control for cases that might be drug-like, but also share feature of toxic compounds.

Cnidaria natural product (NP) molecules were identified from the Marine natural product (NP) database MarinLit (http://pubs.rsc.org/marinlit, accessed in December 2018). Metabolites that were obtained from virus/microbes/bacteria associated with collected Cnidaria were removed from the dataset. The drug data were retrieved from Drugbank (https://www.drugbank.ca/, [34,35,36]) versions 5.1.0 in May 2018. The relevant compounds for comparison with NPs are small organic molecules, so only these were included. The list of drugs included approved and withdrawn molecules. If molecules were withdrawn for toxicity or side effect reasons, they were not included here. However, if they were withdrawn because a newer, more efficient molecule replaced it or because the producing pharmaceutical company shutdown, then these withdrawn drugs were retained. The approved drugs were also filtered to exclude contrasting, dying, or fluorescent agents (e.g., Gadofosvet), metal containing compounds (e.g., Merbromin), and approved additives (e.g., xanthan gum) as none of these molecules are relevant to natural product comparisons. The toxicant data were gathered from the Toxin and Toxin Target Database (T3DB, http://www.t3db.ca/, [37,38]) in June 2019. Drugs (except those withdrawn for toxicity) as well as inorganic molecules were removed from the toxin dataset. Individual metabolites from Marinlit are referred to using their reference codes in the database (e.g., L15811) as many do not have an agreed name.

All drug, Cnidaria, and toxic compounds were downloaded with 2D structures. Physical, chemical, and a small number of absorption-distribution-metabolism-excretion (ADME) properties were defined for each compound using Schrödinger QikProp (51 variables in total, Table S1). LigPrep 4.0 [39] was used to pre-process and minimise all compounds according to the OPLS3 force field before generating variables with QikProp 5.0. If a compound had multiple neutral structures predicted by LigPrep, all resulting ADME properties were averaged. Some variables are used for drug likeness, but not generated by QikProp: the number of rotatable bonds and the octanol-water partition coefficient. Values for these two variables were obtained by submitting drug SMILES codes to Chemicalize -ChemAxon (https://chemicalize.com/) for the number of aromatic rings and SwissADME (http://www.swissadme.ch/ [40]) for the number of structural (Brenk) Alerts (e.g., potentially mutagenic nitro groups, [41]). The drug SMILES codes were downloaded from Drugbank. Comparisons were based on properties generated for 2009 drugs, 2012 toxicants, and 5600 Cnidaria NP.

### 4.2. Drug Likeness Approaches

Comparisons of drugs, toxicants, and NPs were made by calculating a panel of drug likeness scores for each group. This involved the quantitative estimate of drug-likeness (QED, [13]), the relative drug likelihood (RDL, [42], the rule-of-five (Ro5, [7]) and the ADMET-score [16]. The QED [13] is defined using eight drug property histograms: for molecular mass (M_r_), octanol:water partition coefficient (ALOGP), number of hydrogen bond donors (HBDs), number of hydrogen bond acceptors (HBAs), molecular polar surface area (PSA), number of rotatable bonds (ROTBs), number of aromatic rings (AROMs) and number of structural (Brenk) alerts (ALERTS). Peaks in the histograms represent desirable properties for drugs (as they are common), whereas areas falling outside peaks represent undesirable properties. The QED for a molecule is the geometric mean of individual desirability scores based on the molecule’s properties. As the desirability scores for each separate molecular property lie between 0 and 1, the QED for a molecule also lies within this range, with a score close to 0 indicating a lack of similarity to the properties of drugs, and a score close to 1 indicating a molecule similar to most common property values in the reference data set of drugs. 

The relative drug likelihood (RDL, [42]) was calculated using the same eight molecular properties as the QED. The difference between the likeness measures is that the RDL is based on Bayesian estimates of the relative likelihood that a molecule is a drug. For example, a particular molecular weight may be common in drugs and less commonly found in a reference set of compounds (the non-drug toxins as an additional reference in the current study). This situation would lead to a higher relative likelihood that a molecule is a drug at the particular molecular weight. The RDL is calculated for each molecule from the geometric mean of relative likelihoods derived across the eight molecular properties. Values closer to 1 indicate greater drug similarity. The rule-of-five score (Ro5) is based on the thresholds for oral drugs defined by [7]: logarithm of the octanol:water partition coefficient (log P) < 5, molecular weight < 500 Da, number of H-bond donors (HBDs) < 5 and number of H-bond acceptors (HBAs) < 10. A molecule falling within all the thresholds has a score of 0 and would be the most druglike in Ro5 terms, with the maximum score (least druglike) being 4.

In contrast to drug-likeness scores based on molecular properties, the ADMET-score is based on 18 variables (e.g., AMES mutagenicity, acute oral toxicity) predicted from QSAR structure–activity relationships [16]. Admet variables for each compound were generated using the web server admetSAR 2.0 (http://lmmd.ecust.edu.cn/admetsar2/ [43,44]). The final ADMET-score is a weighted average of whether the 18 variables are predicted to be positive/beneficial (coded as 1) or negative/harmful (coded as 0). Two of the weights were unchanged from [16]: the performance of the QSAR model and the relative importance of the endpoint in overall ADMET properties. One weight is based on the frequency of a specific property in the reference drug dataset. The drug data used in the current paper (2009 drugs) differs slightly from that of [16] (1124 drugs), so the weightings used are slightly different (w_1_ in [16]). For Cnidaria and toxic compounds, we used the same w_1_ obtained for drugs and standardised (range 0–1) the data using the minimum and maximum values obtained for drug compounds.

### 4.3. Drug Similarity Approaches

A 2D representation of the relationships among drugs, NPs, and toxicants can be generated using a principal component analysis (PCA, e.g., [45,46]). PCA is a data reduction technique that reprojects multivariate data with the aim of describing the variability in the dataset using a limited number of dimensions. The 51 chemical descriptor variables generated by QikProp had different ranges. Before PCA, data were standardised by subtracting the drug minimum value and dividing by the difference between the drug minimum and drug maximum values [47], so that values fell between 0 and 1. The same drug values were used to rescale and standardise the toxicant and Cnidarian ADME properties. Points in the PCA represent individual drugs, toxins, and NPs. 

In theory, points close together in the PCA represent molecules with similar properties. A 2D PCA may, however, only explain part of the variation among points. This separation can be examined without the restriction of a 2D visualisation using a statistical test, Permanova [48], which compares the observed separation between groups to the patterns generated from random permutation among groups. Permanova was carried out using the adonis function of the vegan [49] R package (999 permutations, method = Euclidian). Measurements of the similarity between pairs of molecules in multivariate space can be made using indices. These similarities were calculated using the Jaccard method in R (vegan package). Each NP had a pairwise Jaccard similarity to each drug, with values towards 0 indicating little similarity in the QikProp variables while maximum similarity occurs at a Jaccard value of 1. Values were summarised as the mean Jaccard drug similarity for each metabolite, representing the separation between a Cnidaria NP and the centre of the drug cloud in multivariate space. 

As an alternative to multivariate summaries of patterns in the 51 QikProp variables, the similarity of molecules can be summarised using molecular fingerprints or common molecular substructures. Fingerprints are a series of binary digits representing the presence or absence of particular sub-structures in a molecule. Fingerprints of drug and Cnidaria molecules were computed from their sdf files (sdf2ap and desc2fp function of ChemmineR package, [50]). Some compounds could not have their fingerprint generated this way and we used a reduced dataset for the analysis: 1965 drugs and 5416 Cnidaria. Fingerprint Tanimoto similarities between drug and Cnidaria fingerprints were computed with the fpSim function of ChemmineR. The Tanimoto similarity is 0 if no substructures in the fingerprint are in common between molecules and 1 if all substructures are in common. Alongside the fingerprint approach, a Tanimoto similarity based on maximum common sub-structures (MCS) was generated for all pairs of Cnidaria NP and drugs. MCS is a graph-based similarity concept defined as the largest sub-graph shared among two molecules. As such MCS differs from structural feature list strategies like fingerprints [50]. The Tanimoto similarity based on maximum common sub-structures (MCS) was computed from the sdf files of drug and Cnidaria compounds using the fmcsR function (fmcsR package, [51]). The two Tanimoto similarities were combined into an index as

$$Tanimoto\;index=\sqrt{\;{\left(fingerprint\;Tanimoto\right)}^{2}+{\left(MCS\;Tanimoto\right)}^{2}}$$

where *fingerprint Tanimoto* is the similarity based on molecular fingerprints and *MCS Tanimoto* is the index based on maximum common substructures. As with the Jaccard similarities, paired Tanimoto index values between Cnidaria NPs and drugs were averaged for each Cnidaria NP to provide an overall similarity to the chemical features of the reference drug set.

### 4.4. Ligand Similarity and Docking

An alternative to comparisons of NPs with drug properties is to conduct virtual screens with ligands of interest or to dock metabolites with drug target proteins. An initial screen was carried out using the BindingDB database, a public web-accessible database of measured binding affinities [52]. The ‘find my compound target’ tool was used to generate a list of targets based on the principle that similar compounds (here Cnidaria NPs and BindingDB ligands) tend to bind to the same proteins. BindingDB ligands are small, drug-like molecules (lead compounds). BindingDB reports based on fingerprint Tanimoto similarities above 0.7 between test compound and ligand. We used the number of targets identified as an index of the drug potential of a NP molecule. 

A more focussed screening for potential cancer activity was carried out using computational docking (Glide in Schrödinger). Target sites from BindingDB were used to generate a screening panel (Table 4). The RCSB Protein Data Bank (www.rcsb.org, PDB) and the Universal Protein Resource (UniProt, https://www.uniprot.org/) were used to obtain 20 targets that were similar to those from BindingDB. Schrödinger’s Maestro 11.9 software was utilised to perform ligand preparation, protein preparation, and receptor grid generation for each target. With the assistance of Glide’s Ligand Docking tool, the entire Cnidaria NP set of metabolites was virtually docked using rounds of high-throughput virtual screening (HTVS), standard precision (SP), and extra precision (XP). Glide scores (XP GScores) are a unitless approximation of the ligand binding free energy derived from a number of terms that include electrostatic attraction and various interactions that can influence binding affinity. Glide scores below −7 were used to identify natural products of potential interest for activity against the screening panel of target proteins.

Serum albumin (P02768) binding affinity may seem an unlikely target property of drugs. However, serum proteins may be effective vectors for the delivery of therapeutics to solid tumours [53] and interactions with albumin can extend drug half-life and aid intracellular delivery [54].

## Figures and Tables

**Table 1:** Correlations of QikProp variables with the first two principal component analysis (PCA) axes and associated contributions. Correlations (Cor.) indicate the degree and type of association between a variable and the PCA axis. Contributions (Contrib., %) indicate the variance due to a single variable compared to that of all the variables combined. Note that only variables with a % contribution superior to the expected average contribution for a Qikprop variable are reported here. This expected average contribution is 100/51 = 1.96%.

PC1			PC2		
Variable	Cor.	Contrib.	Variable	Cor.	Contrib.
RuleOfFive	0.100	3.17	HumanOralAbsorption	0.386	54.73
X.nonHatm	0.095	2.90	glob	0.113	4.67
QPpolrz	0.090	2.60	WPSA	−0.092	3.11
volume	0.088	2.48	X.stars	−0.106	4.14
X.rtvFG	0.085	2.32	RuleOfFive	−0.116	4.92
RuleOfThree	0.085	2.29	RuleOfThree	−0.129	6.15
FOSA	0.084	2.23	QPPCaco	−0.156	8.92
X.noncon	0.080	2.03	QPPMDCK	−0.163	9.80
HumanOralAbsorption	−0.131	5.44			
%HumanOralAbsorption	−0.136	5.90			
QPPMDCK	−0.204	13.22			
QPPCaco	−0.222	15.71			
CNS	−0.259	21.35			

**Table 2:** Differences among median drug-likeness indices for drugs, Cnidaria NPs and toxins. Significance difference among groups tested with a Kruskal–Wallis (KW) test, with pairwise comparisons using Wilcoxon rank sum tests.

Drug-Likeness Index	KW Chi-Squared	df	*p*	Significantly Different Pairs
QED (high values imply drug like)	4648.5	2	<0.001	Drug > Cnidaria
				Drug > Toxic
				Toxic > Cnidaria
RDL (high values imply drug like)	1035.6	2	<0.001	Drug > Cnidaria
				Toxic > Drug
				Toxic > Cnidaria
Ro5 (number of exceptions)	57.7	2	<0.001	Cnidaria > Drug
				Toxic > Drug
ADMET-score (high values imply low predicted toxicity)	1193.7	2	<0.001	Cnidaria > Drug
				Drug > Toxic
				Cnidaria > Toxic

**Table 3:** Pearson’s correlations for Cnidaria NPs between the similarity indices indicating relative positions in chemicals space (Jaccard, Tanimoto), the drug-likeness indices (QED, RDL, Admet Score and Rule-of-five), and the main PCA axes (PC1, PC2). Correlations for which the coefficient shows a moderate to strong correlation (>0.4) are shaded in grey. Rows starting with ‘R’ show the value of the correlation coefficient, with the probability for the observed value indicated by ‘p’. No *p*-value is appropriate for the Tanimoto-Tanimoto correlation, indicated by ‘x’.

		Tanimoto	QED	RDL	Admet Score	Rule-of-five	PC1	PC2
Jaccard	R	0.016	−0.134	0.483	0.291	−0.740	−0.507	0.778
	p	0.239	<0.001	<0.001	<0.001	<0.001	<0.001	<0.001
Tanimoto	R	1.000	0.025	−0.038	−0.077	−0.040	−0.019	−0.159
	p	x	0.064	0.005	<0.001	0.003	0.153	<0.001

**Table 4:** Target proteins used in Glide screening.

Entry	Target Name	UniProID	PDB ID
1	Mitogen-activated protein kinase kinase kinase MLT	Q9NYL2	6JUU
2	Mast/stem cell growth factor receptor Kit	P10721	6HH1
3	Amidophosphoribosyltransferase	Q06203	6CZF
4	Tyrosine-protein kinase BTK	Q06187	6AUB
5	Receptor-type tyrosine-protein kinase FLT3	P36888	4XUF
6	LIM domain kinase 1	P53667	5NXC
7	Platelet-derived growth factor receptor alpha	P16234	5GRN
8	Tyrosine-protein kinase ABL1	P00519	4WA9
9	Thyroid hormone receptor alpha	P10827	4LNW
10	Histone deacetylase 3	O15379	4A69
11	Mitogen-activated protein kinase 14	Q16539	3UVR
12	Alpha-1-acid glycoprotein 1	P02763	3KQ0
13	Vascular endothelial growth factor receptor 1	P17948	3HNG
14	G-protein coupled estrogen receptor 1	Q99527	2R6Y
15	Histone deacetylase 8	Q9BY41	1T69
16	Matrix metalloproteinase-16	P51512	1RM8
17	Thyroid hormone receptor beta	P10828	1Q4X
18	Serum albumin	P02768	4LA0
19	Sex hormone-binding globulin	P04278	1D2S
20	Glucocorticoid receptor	P04150	6DXK

## Data Availability

The manuscript is based on molecules selected from MarinLit (http://pubs.rsc.org/marinlit), Drugbank (https://www.drugbank.ca/) and the Toxin and Toxin Target Database (T3DB, http://www.t3db.ca/). A file with drug likeness values and QikProp properties is at https://doi.org/10.6084/m9.figshare.17697989.

## Supplementary Materials

The following supporting information can be downloaded at: https://www.mdpi.com/article/10.3390/md20010042/s1, Table S1: QikProp descriptors and properties generated for molecules.
