# Supplementary material for: Not Drug-like, but Like Drugs: Cnidaria Natural Products
Source: Mar Drugs. 2021 Dec 30;20(1):42. doi: 10.3390/md20010042 (PMC8779300; doi:10.3390/md20010042)
Supplement: Supplementary file 1 [file marinedrugs-20-00042-s001.zip › marinedrugs-1517098-supplementary.pdf]

SUPPLEMENTARY INFORMATION

Table S1. QikProp descriptors and properties generated for molecules.

Property	Description	Range of values in Cnidaria metabolites
#stars	Number of properties falling outside the 95% range of values for known drugs. Based on the following variables MW, dipole, IP, EA, SASA, FOSA, FISA, PISA, WPSA, PSA, volume, #rotor, donorHB, accptHB, glob, QPpolrz, QPlogPC16, QPlogPoct, QPlogPw, QPlogPo/w, logS, QPLogKhsa, QPlogBB, #metabol	0 to 15
#amine	Number of non-conjugated amine groups	0 to 2
#amidine	Number of amidine and guanidine groups	0 to 2
#acid	Number of carboxylic acid groups	0 to 2
#amide	Number of non-conjugated amide groups	0 to 6
#rotor	Number of non-trivial (not CX3), non-hindered (not alkene, amide, small ring) rotatable bonds	0 to 46
#rtvFG	Number of reactive functional groups	0 to 10
CNS	Predicted central nervous system activity on a –2 (inactive) to +2 (active) scale.	-2 to 2
Mol_MW	Molecular weight of molecule	126.1 to 1056.6
dipole	Computed dipole moment of the molecule	0 to 22.4
SASA	Total solvent accessible surface area (SASA) in square angstroms using a probe with a 1.4 A° radius	306.9 to 1653.7
FOSA	Hydrophobic component of the SASA (saturated carbon and attached hydrogen)	0 to 1502.3
FISA	Hydrophilic component of the SASA (SASA on N, O, and H on heteroatoms)	0 to 421.3
PISA	π (carbon and attached hydrogen) component of the SASA	0 to 383.2
WPSA	Weakly polar component of the SASA (halogens, P, and S)	0 to 319.6
Volume	Total solvent-accessible volume in cubic angstroms using a probe with a 1.4 A° radius	457.5 to 3101.3
donorHB	Estimated number of hydrogen bonds that would be donated by the solute to water molecules in an aqueous solution. Values are averages taken over a number of configurations, so they can be non-integer	0 to 9.5
accptHB	Estimated number of hydrogen bonds that would be accepted by the solute from water molecules in an aqueous solution.	0 to 25.5
Dip^2/V	Square of the dipole moment divided by the molecular volume. This is the key term in the Kirkwood-Onsager equation for the free energy of solvation of a dipole with volume V	0 to 0.273
ACxDN^.5/SA	Index of cohesive interaction in solids. This term represents the relationship (accptHB.sqrt(donorHB))/(SA)	0 to 0.074

glob	Globularity descriptor, $(4\pi r^2)/(SASA)$, where r is the radius of a sphere with a volume equal to the molecular volume. Globularity is 1.0 for a spherical molecule	0.607 to 0.997
QPpolrz	Predicted polarizability in cubic angstroms	12.2 to 95.8
QPlogPC16	Predicted hexadecane/gas partition coefficient.	4.38 to 34.05
QPlogPoct	Predicted octanol/gas partition coefficient.	5.9 to 51.4
QPlogPw	Predicted water/gas partition coefficient	-1.26 to 32.86
QPlogPo/w	Predicted octanol/water partition coefficient	-2.03 to 13.55
QPlogS	Predicted aqueous solubility, log S. S in mol dm^{-3} is the concentration of the solute in a saturated solution that is in equilibrium with the crystalline solid.	-16.13 to 1.33
CIQPlogS	Conformation-independent predicted aqueous solubility, log S. S in mol dm^{-3} is the concen- tration of the solute in a saturated solution that is in equilibrium with the crystalline solid	-16.13 to 0.27
QPlogHERG	Predicted IC_{50} value for blockage of HERG K^+ channels	-7.9 to 3.2
QPPCaco	Predicted apparent Caco-2 cell permeability in nm/sec. Caco-2 cells are a model for the gut- blood barrier. QikProp predictions are for non-active transport	0.092 to 9906
QPlogBB	Predicted brain/blood partition coefficient. Note: QikProp predictions are for orally delivered drugs so, for example, dopamine and serotonin are CNS negative because they are too polar to cross the blood-brain barrier	-7.5 to 1.6
QPPMDCK	Predicted apparent MDCK cell permeability in nm/sec. MDCK cells are considered to be a good mimic for the blood-brain barrier. QikProp predictions are for non-active transport	0.07 to 10000
QPlogKp	Predicted skin permeability, log K_P	-11.2 to 2.3
IP(ev)	PM3 calculated ionization potential	2.7 to 11.2
EA(eV)	PM3 calculated electron affinity	-3.1 to 2.9
#metab	Number of likely metabolic reactions.	0 to 20
QPlogKhsa	Prediction of binding to human serum albumin	-2.1 to 3.7
HumanOralAb- sorption	Predicted qualitative human oral absorption: 1, 2, or 3 for low, medium, or high. Based on rule-based thresholds in relevant variables	1 to 3
PercentHuman- OralAbsorption	Predicted human oral absorption on 0 to 100% scale.The prediction is based on a quantita- tive multiple linear regression model.	0 to 100
SAFluorine	Solvent-accessible surface area of fluorine atoms	0 to 0
SAamideO	Solvent-accessible surface area of amide oxygen atoms	0 to 73.1
PSA	Van der Waals surface area of polar nitrogen and oxygen atoms	0 to 317.2

#NandO	Number of nitrogen and oxygen atoms	0 to 22
RuleOfFive	Number of violations of Lipinski's rule of five	0 to 4
RuleOfThree	Number of violations of Jorgensen's rule of three	0 to 36
#ringatoms	Number of atoms in rings	0 to 10
#in34	Number of atoms in 3- or 4-membered rings	0 to 32
#in56	Number of atoms in 5- or 6-membered rings	0 to 26
#noncon	Number of ring atoms not able to form conjugated aromatic systems	9 to 74
#nonHatm	Number of heavy atoms (nonhydrogen atoms)	0 to 3
Jm	Predicted maximum transdermal transport rate, K_p × MW × S (μg cm^{-2} hr^{-1}). Kp and S are obtained from the aqueous solubility and skin permeability, QPlogKp and QPlogS.	0 to 249048
